# A Comprehensive Update on the Bioactive Compounds from Seagrasses

**DOI:** 10.3390/md20070406

**Published:** 2022-06-21

**Authors:** Christina Mutiara Putri Gono, Peni Ahmadi, Triana Hertiani, Eris Septiana, Masteria Yunovilsa Putra, Giuseppina Chianese

**Affiliations:** 1 Faculty of Pharmacy, Universitas Gadjah Mada (UGM), Sekip Utara, Yogyakarta 55281, Indonesia; christinamutiara99@mail.ugm.ac.id; 2 Research Center for Vaccine and Drug, Research Organization for Health, National Research and Innovation Agency (BRIN), Jl. Raya Bogor Km. 46, Cibinong 16911, Indonesia; eris002@brin.go.id; 3 Department of Pharmaceutical Biology, Faculty of Pharmacy, Universitas Gadjah Mada (UGM), Sekip Utara, Yogyakarta 55281, Indonesia; hertiani@ugm.ac.id; 4 Department of Pharmacy, School of Medicine and Surgery, University of Naples Federico II, Via D. Montesano 49, 80131 Napoli, Italy

**Keywords:** marine angiosperm, bioactive compound, potential drug, medical properties

## Abstract

Marine angiosperms produce a wide variety of secondary metabolites with unique structural features that have the potential to be developed as effective and potent drugs for various diseases. Recently, research trends in secondary metabolites have led to drug discovery with an emphasis on their pharmacological activity. Among marine angiosperms, seagrasses have been utilized for a variety of remedial purposes, such as treating fevers, mental disorders, wounds, skin diseases, muscle pain, and stomach problems. Hence, it is essential to study their bioactive metabolites, medical properties, and underlying mechanisms when considering their pharmacological activity. However, there is a scarcity of studies on the compilation of existing work on their pharmacological uses, pharmacological pathways, and bioactive compounds. This review aims to compile the pharmacological activities of numerous seagrass species, their secondary metabolites, pharmacological properties, and mechanism of action. In conclusion, this review highlights the potency of seagrasses as a promising source of natural therapeutical products for preventing or inhibiting human diseases.

## 1. Introduction

Marine natural products have attracted the attention of scientists worldwide for the last five decades. Marine organisms are an exceptional reservoir of new bioactive compounds that exhibit a greater variety of structural and chemical features than terrestrial natural products [1]. Several chemically unique compounds from marine organisms with different biological activities have been isolated, and some of them are under investigation for development as new pharmaceuticals [2]. In addition, some marine-derived drugs are approved by the European Medicines Agency (EMA) and/or the Food and Drug Administration (FDA) [3]. Although they are mostly anticancer agents, a number of viral infections, chronic pain relievers, and hypertriglyceridemia drugs have also been approved. However, no drugs isolated from seagrasses have been approved by the FDA or EMA.

Marine organisms, including seagrasses, are valuable sources of biologically active compounds for the treatment of human diseases. Seagrasses are one of the true marine flowering plants that belong to the group of angiosperms, which have 72 species worldwide, divided into four families (Zosteraceae, Hydrocharitaceae, Posidoniaceae, and Cymodoceaceae) [4,5]. Seagrasses are eukaryotic organisms found in shallow water areas of temperate, subtropical, and tropical seas, except in polar regions [6]. Seagrass beds are found in shallow coastal areas around the world, with distribution ranges around 50–90 m in depth [6]. Seagrass meadows can store carbon, improve water quality, provide food and habitat, and act as biological indicators [7]. Seagrasses are known to produce a wide variety of secondary metabolites that act as defense mechanisms under stress conditions. These active metabolites, such as polyphenols, terpenoids, and halogenated compounds, are produced by several species of the seagrass reported to have anticancer (antitumor), antifungal, anti-inflammatory, antimicrobial, antiviral, antidiabetic, antimalarial, antioxidant, anti-aging, and cytotoxic properties. These species are also effective in the prevention of human diseases [8]. Seagrasses have been used in folk medicine for a variety of remedial purposes, such as the treatment of fevers, stomach problems, muscle pain, wounds, and skin diseases; they are also used as a remedy for stings of different kinds of rays and as tranquilizers for babies [9]. In addition to their pharmacological activity, seagrasses have been utilized for making baskets, burned to obtain salt, mattress filling material, thatched roofs, fertilizer, paper materials to transport fragile items, and nitrocellulose, among other uses [10].

Based on the benefits discussed above, this review amalgamates a comprehensive compilation of the phytochemical composition and biomedical applications of seagrasses around the world.

## 2. Methods

Articles published from 2011 to 2022 were retrieved through several databases, namely PubMed, Springer, Elsevier, MDPI, and Google Scholar, for investigating the bioactive compounds and pharmacological activity of marine angiosperms (seagrasses), as well as the compounds isolated from seagrasses under the clinical trial. The search terms used were “Marine angiosperm” OR “Seagrasses” AND “bioactive compounds” OR “phytochemicals” OR “chemical compounds” OR “anticancer” OR “antioxidant” OR “anti-inflammatory” OR “antimicrobial” OR “antibacterial” OR “antifungal” OR “antiviral” OR “anti-dengue” OR “anti-hyperlipidemia” OR “lipid reducing” OR “antidiabetic” OR “hepatoprotective” OR “anti-aging” OR “mechanism of action” OR “underlying mechanism” OR “clinical trial.” The search was restricted to articles published in English and Indonesian languages. From the 138 articles that reported the bioactive compounds, their pharmacological activity, clinical trial, and the underlying mechanism of marine angiosperms were found. A limitation of this study is the bioavailability and pharmacokinetic evaluation of the bioactive compounds, which were not well indicated or mentioned in the original articles reported.

**Figure 1:**
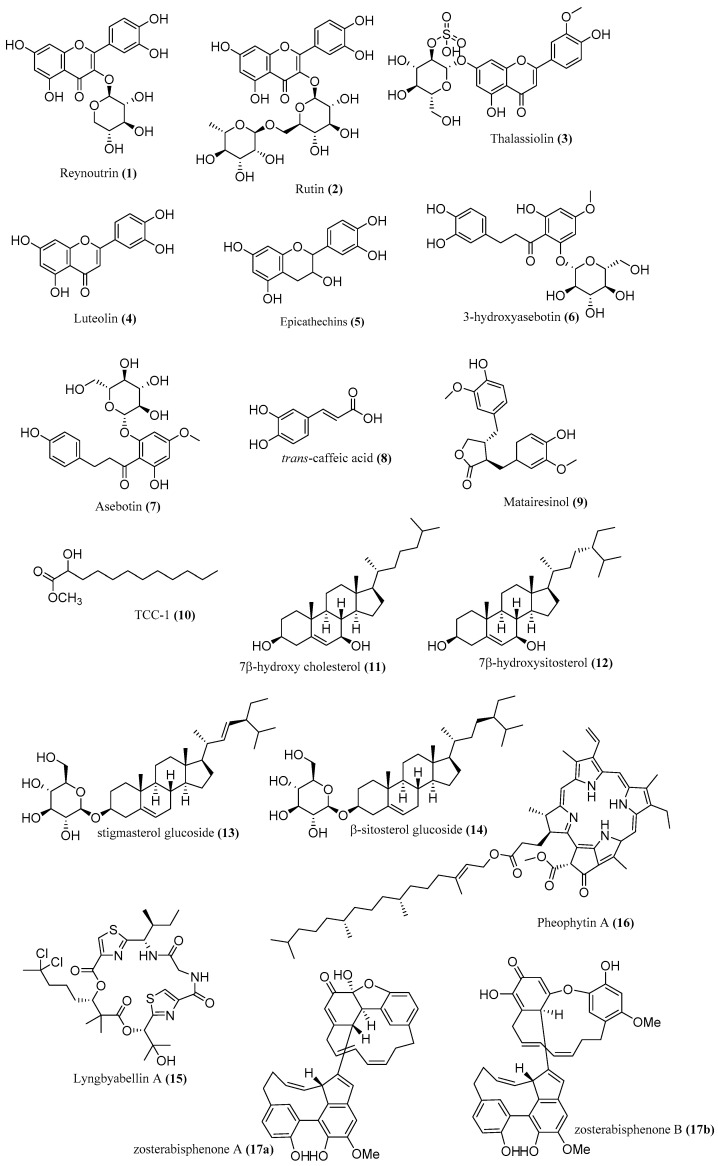
Chemical compounds **1**–**17**.

**Figure 2:**
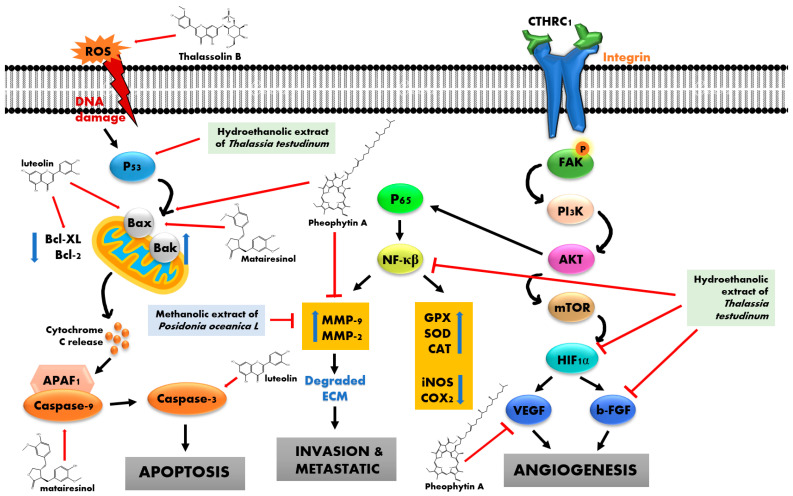
Anticancer mechanism of seagrasses.

**Figure 3:**
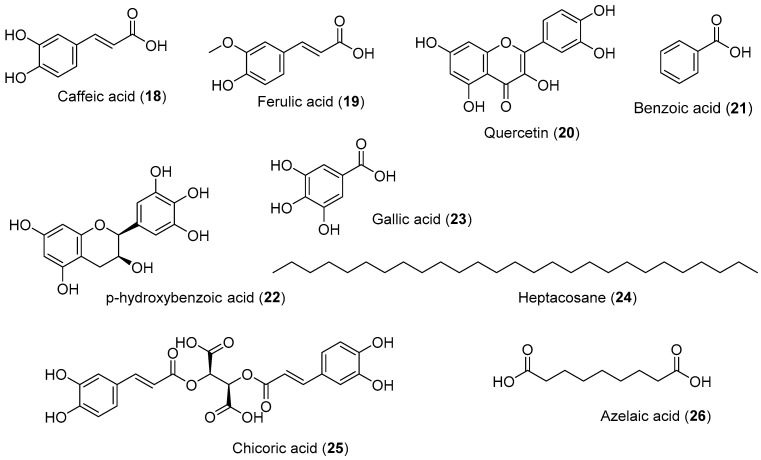
Chemical compounds **18**–**26**.

**Figure 4:**
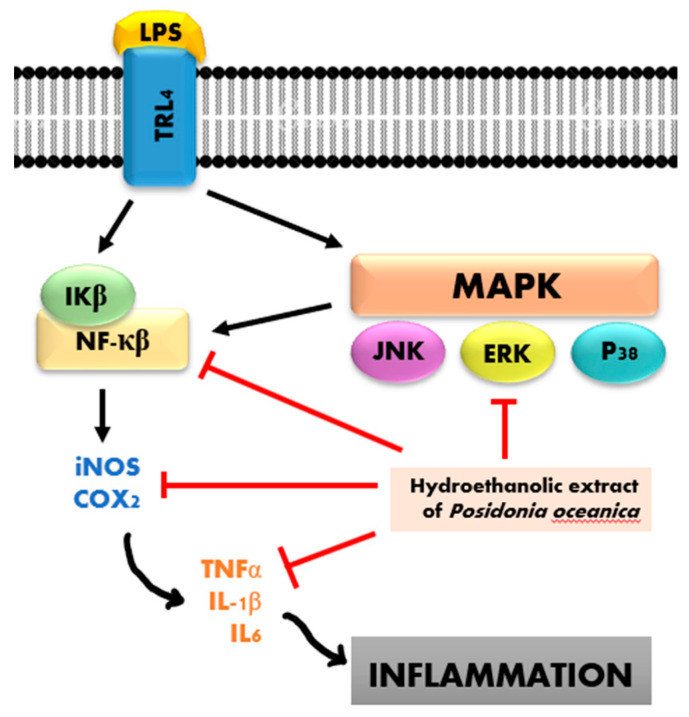
Anti-inflammatory mechanism of *P. oceanica*.

**Figure 5:**
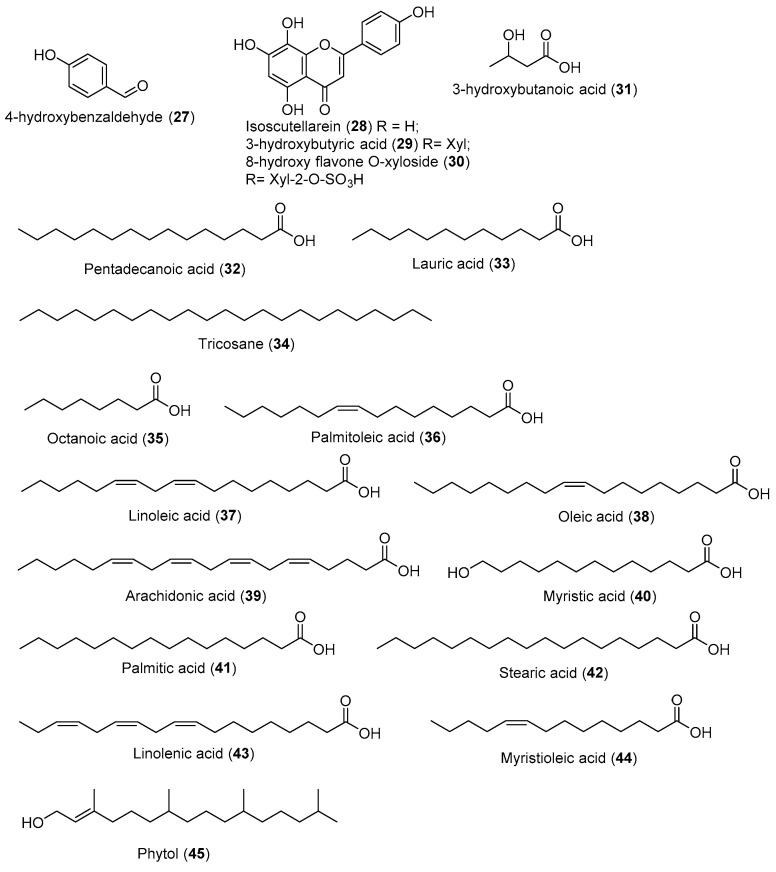
Chemical compounds **27**–**45**.

**Figure 6:**
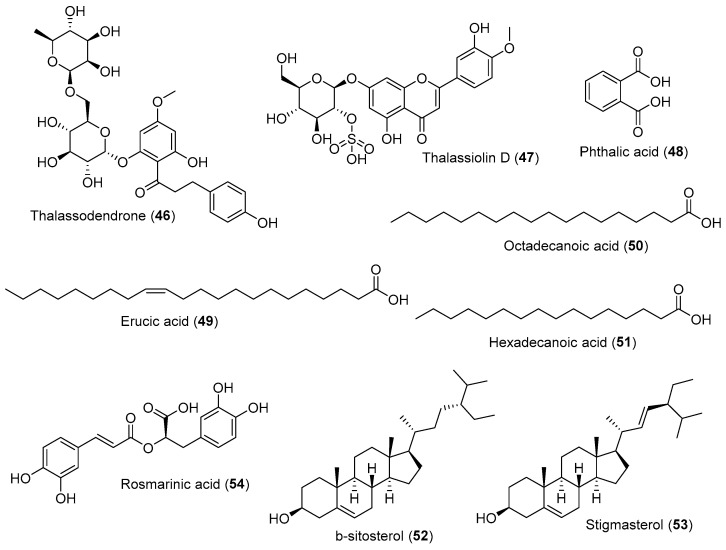
Chemical compounds **46**–**54**.

In the following paragraphs, the seagrass secondary metabolites and extracts are clustered by their bioactivity. Compound structures, ordered by compound class, are depicted in Figures 1–6 in Section 3. Seagrasses species and their family reviewed are listed in Table 1. We have simplified the reading of the text by summarizing the bioactivities of extracts in Tables 2–7 in Section 3, in which the source species are listed in alphabetical order.

**Table 1:** Seagrasses species and their family.

Family	Species
Cymodoceaceae	*Cymodocea nodosa* *Cymodocea rotundata* *Cymodocea serrulata* *Halodule pinifolia* *Halodule uninervis* *Syringodium isoetifolium* *Syringodium filiforme* *Thalassodendron ciliatum*
Hydrocharitaceae	*Enhalus acoroides* *Halophila beccarii* *Halophila ovalis* *Halophila ovata* *Halophila stipulaceae* *Thalassia hemprichii* *Thalassia testudinum*
Posidoniaceae	*Posidonia oceanica*
Zosteraceae	*Zostera marina* *Zostera noltei*

## 3. Bioactivities of Extracts and Compounds Isolated from Seagrasses

### 3.1. Anticancer

Cancer is a complex disease characterized by the over-proliferation of cells due to failures in cellular modulation and the obstruction of cell-cycle progression [11]. It invades and destroys normal cells, creating an imbalance in the body with the possibility of becoming metastatic [12]. Cancer is the leading cause of human mortality worldwide and caused 10 million deaths in 2020 [13]. Current treatments for cancer include radiotherapy, chemotherapy, and chemically derived drugs that have several impacts on healthy cells. There is also the problem of an increase in tumor resistance to current therapeutic agents [11]. Thus, the discovery of new anticancer agents from natural products, especially plants, is under investigation. Medicinal plants represent a good source of discovery and development of anticancer agents. Medicinal plants produce many secondary metabolites, which expand the scope of effective and new drugs for cancer treatment. These metabolites can interfere with a large set of molecular targets in cells such as proteins, DNA, RNA, and the cell membrane [14].

Marine natural products have been found to exhibit anticancer activity in vitro on a wide range of tumor cell lines. In addition, most reports concerning their mechanism of action in inhibiting tumor growth, both in vitro and in vivo, suggest this mechanism is mediated via the apoptosis, necrosis, and lysis of the tumor cells [15]. Various extracts and bioactive compounds (Figure 1) of nine seagrass species were reported to exhibit anticancer activities of hepatoma (HepG2), cervical carcinoma (HeLa), human colorectal carcinoma (HCT116), human osteosarcoma (MG63), breast cancer (MCF-7), etc. (Table 2). These extracts exhibit anticancer activity through antiproliferative, cytotoxic, cytostatic, and antimetastatic action; inducing apoptotic and antioxidative activity; provoking cell-cycle arrest; inhibiting angiogenesis; and reducing cancer cell viability [16].

Reynoutrin (**1**), rutin (**2**), and asebotin (**7**) isolated from methanolic extract of *T. ciliatum* exhibit anticancer activity against HepG2 and HCT116 cells with IC_50_ 7.25 μM, 11.17 μM, 32.76 μM, 20 μM, 8.55 μM, and 14.32 μM, respectively [17]. Moreover, 3-hydroxyasebotin (**6**) isolated from methanolic extracts of *T. ciliatum* showed 50% inhibition against HCT116 cells at 9.77 μM [17]. Trans-caffeic acid (**8**) isolated from methanolic extracts of *T. ciliatum* exhibited anticancer activity against HCT116, HepG2, and HeLa, with IC_50_ 23.03 μM, 17.48 μM, and 6.25 μg/mL [17]. The antitumor activity of zosterabisphenones (**17a**,**b**) from *Z. marina* on the cell lines (HCT116 and HepG2 cells) was evaluated, and zosterabisphenone B (**17**) was found to have a more selective effect on HCT116 cells than HepG2 (IC_50_ 3.6 ± 1.1 μM at 48 h) [18].

TCC-1 (**10**), 7β-hydroxy cholesterol (**11**), 7β-hydroxysitosterol (**12**), stigmasterol glucoside (**13**), and β-sitosterol glucoside (**14**) isolated from methylene dichloride–methanol extract of *T. ciliatum* showed cytotoxic activity against HepG2 and MCF7 cells with an IC_50_ value near 20 μM [19]. Epicatechins (**5**) isolated from methanolic extracts of *T. ciliatum* and hydroalcoholic extracts of *P. oceanica* (1.4%) exhibited cytotoxic activity against MCF7 cells with IC_50_ 102 μg/mL [17]. Luteolin (**4**) isolated from ethyl acetate and methanolic extract of *H. stipulacea* showed 35% inhibition of MG63 cell at 2.5 µg/mL and 72% inhibition of MG64 at 12.5 µg/mL [20,21]. It inhibits the proliferation of MG63 and MG64 cells by increasing the expression of Bax protein as well as down-regulating the expression of BCL-2 and caspase-3 [20,21]. Matairesinol (**9**) isolated from hexane extract of *H. stipulacea* showed 50% inhibition of CCRF-CEM cell at 4.27 µM [16,21]. In addition, it inhibits the proliferation of CCRF-CEM by inducing S phase arrest and apoptosis by enhancing the expression of Bax, caspase-9, and cascade [16,21]. Lyngbyabellin A (**15**) isolated from hexane extracts of *H. stipulacea* exhibited cytotoxicity against HCT116 with IC_50_ 40.9 nM [21,22]. Thalassiolin B (**3**) isolated from the polyphenolic fraction of *T. testudinum* revealed antitumor activity against HCT15 and HT29 cells with IC_50_ 38.75 µg/mL and 121.71 µg/mL, respectively [23]. Pheophytin A (**16**) isolated from ethyl acetate extracts of *S. isoetifolium* displayed an IC_50_ value of 22.9 µM against A549 cells [24]. Compound **16** increased Bax expression, reduced the levels of MMP-2 and VEGF, and bonded to translocation protein (TPSO) with a binding energy of −3.62 kcal/mol [24]. Bioinformatics analysis showed that this extract is classified as class 5 cytotoxicity, which means it is safe to be used at less than 5000 mg/kg. It also follows Lipinski’s rule of five and, thus, can be administered orally [24].

The polyphenol compounds from *T. ciliatum* act as anticancer agents by mobilizing endogenous copper (and possibly chromatin-bound copper) and the result of prooxidant action [17]. Balls and leaves hydroethanolic extract from *P. oceanica* L. inhibit metastatic activity by decreasing the expression of MMP-2 and MMP-9 [25]. The aqueous extract and silver nanoparticles from *C. serrulata* induced the inactivation of replication and, reacting with sulfur-containing proteins, led to the inhibition of enzyme functions, which resulted in the loss of cell viability and cell death [26]. Hydrophilic extracts of *P. oceanica* L. inhibit HT1080 cell migration by decreasing the expression of MMP2 and MMP9 [27]. The chloroform fraction of the hydroethanolic extract from *T. testudinum* inhibits the proliferation and migration of A549 and EA.hy926 cells by decreasing the expression and activity of hypoxia-inducible factor 1 (HIF-1) [28]. Moreover, hydroethanolic extracts from *T. testudinum* suppress the angiogenesis of RKO, SW480, and CT26 by inhibiting bFGF-induced neovascularization, triggering ATF4-P53-NFκB-specific gene expression and autophagy stress pathways, and promoting antitumor immunogenic cell death (IFNγ, PD-1, and ZAP70) [29]. Hydroethanolic extracts from *T. testudinum* also induced cytotoxicity based on oxidative stress, nuclear damage, and sustained hypercalcemia in HepG2 cells [30]. Furthermore, Thalassiolin B from *T. testudinum* increased the production of ROS and induces pro-apoptotic effects on HCT15 and HT29 cells [23]. *T. testudinum* extract up to 1000 μg/mL did not induce significant toxicity effects in normal cells (hepatocyte, lymphocyte, CHO, VERO, 3T3, MDCK, and BHK-21), indicating that it is selective in cancer cells [30]. The anticancer mechanisms of various seagrasses are summarized in Figure 2 [31,32].

**Table 2:** Anticancer activity of seagrasses.

Species	Extract/Active Compound	Cell Line	Inhibition	References
*C. serrulata*	AgNPs (silver nanoparticles)	HeLa	IC_50_: 34.5 µg/mL	[26]
	Aqueous extract	HeLa	IC_50_: 107.7 µg/mL	[26]
*H. stipulacea*	Ethyl acetate extract (leaves)	MG63	IC_50_: 29.4 μg/mL	[21]
		SHSY5Y	IC_50_: 10.6 μg/mL	[21]
	Ethyl acetate extract (stems)	MG63	IC_50_: 19.1 μg/mL	[21]
		SHSY5Y	IC_50_: 18.7 μg/mL	[21]
	Hexane extract (leaves)	HCT116	IC_50_: 19.5 μg/mL	[21]
	Hexane extract (stems)	HCT116	IC_50_: 7.6 μg/mL	[21]
*P. oceanica*	EtOH/H_2_O (7:3)	SH-SY5Y	Inhibits 57% cell migration at 3 μg/mL after 7 h treatment	[33]
	Hydrophilic extract	HT1080	Inhibits 72.3% cell migration after 12 h treatment	[27]
	MeOH/H_2_O 7:3 (balls extract)	HepG2	IC_50_: 24.3 µg/mL	[25]
		MCF7	IC_50_: 22.6 µg/mL	[25]
		HCT116	IC_50_: 22.5 µg/mL	[25]
	MeOH/H_2_O 7:3 (leaves extract)	HepG2	IC_50_: 17 µg/mL	[25]
		HepG2	IC_50_: 28.3 µg/mL	[25]
		HCT116	IC_50_: 27.8 µg/mL	[25]
*S. filiforme*	Chloroform fraction of hydroethanolic extract	A549	Decreases the viability of A549 cells below 60% at 100 µg/mL	[34]
*T. ciliatum*	Methanolic extract	HCT-116	IC_50_: 4.2 μg/mL	[17]
		HeLa	IC_50_: 9.8 μg/mL	[17]
		HepG2	IC_50_: 8.12 μg/mL	[17]
		MCF7	IC_50_: 4.12 μg/mL	[17]
*T. testudinum*	Chloroform fraction of the hydroethanolic extract	A549	IC_50_: 20.4 µg/mL	[28]
		EA.hy926	IC_50_: 248.4 µg/mL	[28]
	Hydroethanolic extract	RKO	IC_50_: 174.9 µg/mL	[29]
		SW480	IC_50_: 58.9 µg/mL	[29]
		CT26	IC_50_: 115.3 µg/mL	[29]
		HepG2	IC_50_: 102 μg/mL	[30]
		PC12	IC_50_: 135 μg/mL	[30]
		Caco2	IC_50_: 165 μg/mL	[30]
		4T1	IC_50_: 129 μg/mL	[30]
	Polyphenol fraction of hydroethanolic extract	HCT15	IC_50_: 22.47 µg/mL	[23]
		HT29	IC_50_: 93.11 µg/mL	[23]
		HT29	IC_50_: 121.71 µg/mL	[23]

### 3.2. Antioxidant

Oxidative stress is caused by an imbalance between the free radicals and antioxidants in the body that can irreversibly damage several cellular structures. Oxidative stress has been recognized as being involved in the pathology of many age-related diseases, such as atherosclerosis, diabetes, neurodegenerative diseases, aging, and cancer [35]. Antioxidants from endogenous and exogenous sources may help to counteract the negative effects of oxidative stress. The most effective and widely used strategy to reduce oxidative stress is exogenous antioxidants supplementation [36]. In recent years, there have been concerns over the safety of synthetic antioxidants. Therefore, antioxidants from natural sources are attracting more attention. Natural products, such as carotenoids, tocopherols, and flavonoids, are well recognized as strong antioxidants with activity in scavenging free radicals and relieving cellular damage caused by oxidation [37]. Another group of naturally-derived chemicals, polysaccharides, has also attracted wide attention because of their promising in vitro and in vivo biological activity [38].

Marine organisms have been considered a promising source of nutrients and bioactive compounds. In recent years, many polysaccharides from marine organisms with antioxidant activity have been isolated and identified, but the characteristics of these polysaccharides have rarely been summarized, and their structure–activity relationships have been scarcely reported [38]. Seagrasses are known to produce secondary metabolites as defense mechanisms under stress conditions, and these compounds are found to be anti-oxidative (Figure 3). The methanol extract of *H. ovalis* at 500 µg/mL had higher reducing power than ascorbic acid [39]. The superoxide dismutase activity of *E. acoroides* showed that the ethyl acetate extract was the most active, with an IC_50_ value of 7 ppm; quercetin and catechin, as reference compounds, had IC_50_ values of 5 and 13 ppm, respectively [40]. Old leaf extracts of *H. stipulacea* induced a 3.9-fold up-regulation of the CYBB gene and the down-regulation of EPHX2 (19-fold), EPX (2-fold), MBL2 (11.6-fold), MPO (6.9-fold), and SPINK1 (10-fold) genes in WI-38 cells treated with 10 mM of H_2_O_2_. It is indicated that the oxidative stress response was not activated when cells were treated with *H. stipulacea* [41]. Moreover, WI-38 cells pre-treated with old leaf extracts of *H. stipulacea* before an injury with 10 mM of H_2_O_2_ exhibited an up-regulation of genes involved in the antioxidant cell response, such as glutathione peroxidase 5/GPX5 (2.3-fold), keratin 1/KRT1 (2.2-fold), lactoperoxidase/LPO (2.6-fold), metallothionein 3/MT3 (2.0-fold), NADPH oxidase 5/NOX5 (2.8-fold), and thyroid peroxidase/TPO (2.3-fold) [41]. A recent comparative study between *Z. marina* and *Z. notei* ethyl acetate extracts displayed the antioxidant activity of both extracts ascribed in *Z. marina* fraction to rosmarinic acid. On the other hand, the *Z. noltei* extract had the capacity to chelate copper and iron ions, suggesting its potential application to alleviate Alzheimer’s disease (AD) symptoms [42] (Table 3).

Caffeic acid (**18**) isolated from chloroform fractions of *S. filiforme* (0.05%) and methanolic extracts of *T. ciliatum* showed 50% inhibition at 3.5 mM in DPPH assay [17]. Compounds **1**, **6**, and **2**, exhibited 50% DPPH radical at 1.63 mM, 1.62 mM, and 0.99 mM [17]. Compound **5** led to a potent decrease in GPx levels and a significant increase in SOD levels [43]. Ferulic acid (**19**) isolated from hydroalcoholic acid extracts of *P. oceanica* (1.7%) and methanolic extracts of *T. ciliatum* formed stable phenoxyl radicals, bonded to transition metals, and reduced ROS production [44]. Quercetin (**20**) isolated from hydrochloric acid extracts of *P. oceanica* and chloroform fractions of *S. filiforme* (0.13%) inhibited DPPH radicals, with IC_50_ 5.5 µg/mL [45]. Benzoic acid (**21**) isolated from methanolic extracts of *H. ovalis* (11.11%), as well as chloroform fractions of *S. filiforme* (1.12%) and *T. testudinum* (0.14%), neutralized superoxide radicals [46]. Further, p-Hydroxybenzoic acid (**22**) isolated from chloroform fraction of *S. filiforme* (2.57%) and *T. testudinum* (0.55%) showed radical scavenging activity of DPPH radicals [47] Gallic acid (**23**) isolated from hydroethanolic extracts of *P. oceanica* (0.4%) revealed radical scavenging activity and was able to enhance the enzymatic antioxidant, including catalase (CAT), glutathione S transferase (GST), glutathione (GSH), and glutathione peroxidase (GPx) [48]. Heptacosane (**24**) isolated from chloroform fractions of *S. filiforme* (1.53%) and ethyl acetate extracts of *E. acoroides* (4.17%) exhibited potent antioxidant activity [49]. Chicoric acid (**25**) isolated from ethanolic extracts of *P. oceanica* inhibited DPPH radicals, with IC_50_ 23 µg/mL [44]. Azelaic acid (**26**) isolated from chloroform fractions of *S. filiforme* (5.06%) and *T. testudinum* (0.96%) demonstrated potent antioxidant properties [50].

**Table 3:** Antioxidant activity of seagrasses.

Species	Extract/Active Compound	% Inhibition	Assay	References
*C. nodosa*	Sulfated polysaccharide	OD: 0.3 mm at 1 mg/mL	FRAP	[51]
		82.44% inhibition at 0.5 mg/mL	DPPH	[51]
		82.6% inhibition at 2 mg/mL	ABTS	[51]
*C. rotundata*	Aqueous methanol (1:4)	70.30%	DPPH	[52]
		53.74%	Hydroxyl radical scavenging activity	[52]
	Ethyl acetate extract	50% inhibition at 362.56 ppm	DPPH	[53]
	Methanolic extract	50% inhibition at 214.68 ppm	DPPH	[53]
*C. serrulata*	Aqueous extract	65.68% inhibition at 100 μg/mL	DPPH	[26]
	Aqueous fraction	53.8% inhibition at 600 µg/mL	DPPH	[54]
	Aqueous methanol (1:4)	41.28%	DPPH	[52]
	Butanol fraction	82.6% inhibition at 600 µg/mL	DPPH	[54]
	Ethanolic extract	28.423 mg Gallic acid/g	FRAP	[55]
		61.85%	DPPH	[55]
	Ethyl acetate fraction	89.45% inhibition at 600 µg/mL	DPPH	[54]
	Petroleum ether fraction	26.75% inhibition at 600 µg/mL	DPPH	[54]
	Silver nanoparticles	87.99% inhibition at 100 μg/mL	DPPH	[26]
*E. acoroides*	Aqueous extract	30.68% inhibition at 200 µg/mL	DPPH	[56]
		83.67% inhibition at 200 µg/mL	ABTS	[56]
		44.91% inhibition at 200 µg/mL	SO assay	[56]
		56.64% inhibition at 200 µg/mL	NO assay	[56]
		0.42% inhibition at 200 µg/mL	FRAP	[56]
	Aqueous fraction	15.8% inhibition at 600 µg/mL	DPPH	[54]
	Aqueous methanol (1:4)	35.80%	DPPH	[52]
	Butanol fraction	19.4% inhibition at 600 µg/mL	DPPH	[54]
	Chloroform extract	32.92% inhibition at 200 µg/mL	DPPH	[56]
		60.52% inhibition at 200 µg/mL	ABTS	[56]
		52.18% inhibition at 200 µg/mL	SO assay	[56]
		22.6% inhibition at 200 µg/mL	NO assay	[56]
		0.21% inhibition at 200 µg/mL	FRAP	[56]
	Ethanolic extract	30% inhibition at 200 µg/mL	DPPH	[56]
		42.93% inhibition at 200 µg/mL	ABTS	[56]
		36.94% inhibition at 200 µg/mL	SO assay	[56]
		39.7% inhibition at 200 µg/mL	NO assay	[56]
		0.17% inhibition at 200 µg/mL	FRAP	[56]
		3.373 mg gallic acid/g	FRAP	[55]
		24.13%	DPPH	[55]
	Ethyl acetate extract	50% inhibition at 153.4 ppm	DPPH	[53]
		30.72% inhibition at 200 µg/mL	DPPH	[56]
		78.31% inhibition at 200 µg/mL	ABTS	[56]
		44.56% inhibition at 200 µg/mL	SO assay	[56]
		29.33% inhibition at 200 µg/mL	NO assay	[56]
		0.21% inhibition at 200 µg/mL	FRAP	[56]
	Ethyl acetate fraction	80.57% inhibition at 600 µg/mL	DPPH	[54]
	Hexane extract	26.88% inhibition at 200 µg/mL	DPPH	[56]
		61.43% inhibition at 200 µg/mL	ABTS	[56]
		42.7% inhibition at 200 µg/mL	SO assay	[56]
		25.98% inhibition at 200 µg/mL	NO assay	[56]
		0.21% inhibition at 200 µg/mL	FRAP	[56]
	Methanolic extract	50% inhibition at 115.79 ppm	DPPH	[53]
		70.2 mg Trolox equivalents (TE)/g DM	ORAC	[57]
	Petroleum ether fraction	33.75% inhibition at 600 µg/mL	DPPH	[54]
*H. beccarii*	Aqueous fraction	24.4% inhibition at 600 µg/mL	DPPH	[54]
	Aqueous fraction of aqueous methanol 1:1 extract	IC_50_: 31.8 µg/mL	DPPH	[45]
	Butanol fraction	13.9% inhibition at 600 µg/mL	DPPH	[54]
	Ethyl acetate fraction	84.56% inhibition at 600 µg/mL	DPPH	[54]
	Petroleum ether fraction	14.33% inhibition at 600 µg/mL	DPPH	[54]
*H. ovalis*	Acetone extract	73.55%	DPPH	[58]
		23.58%	Hydrogen peroxide scavenging activity	[58]
	Aqueous fraction	5.2% inhibition at 600 µg/mL	DPPH	[54]
	Butanol fraction	12.2% inhibition at 600 µg/mL	DPPH	[54]
	Ethanolic extract	12.042 mg gallic acid/g	FRAP	[55]
	Ethyl acetate fraction	6.68% inhibition at 600 µg/mL	DPPH	[54]
	Hexane extract	8.20%	DPPH	[58]
		21.21%	DPPH	[55]
	Methanolic extract	IC_50_: 0.13 mg/mL	DPPH	[39]
		IC_50_: 0.65 mg/mL	Superoxide radicals scavenged	[39]
		72.5 mg Trolox equivalents (TE)/g DM	ORAC	[57]
	Petroleum ether fraction	4.77% inhibition at 600 µg/mL	DPPH	[54]
*H. ovata*	Ethanolic extract	5.856 mg gallic acid/g	FRAP	[55]
		16.93%	DPPH	[55]
*H. pinifolia*	Acetone extract	66.98%	DPPH	[58]
		10.63%	NO scavenging activity	[58]
	Aqueous fraction	22.2% inhibition at 600 µg/mL	DPPH	[54]
	Aqueous methanol (1:4)	58.60%	DPPH	[52]
		51.05%	hydroxyl radical scavenging activity	[52]
	Butanol fraction	28.4% inhibition at 600 µg/mL	DPPH	[54]
	Ethanolic extract	42.611 mg gallic acid/g	FRAP	[55]
		68.07%	DPPH	[55]
	Ethyl acetate fraction	80.25% inhibition at 600 µg/mL	DPPH	[54]
	Hexane extract	68.64%	Hydrogen peroxide scavenging activity	[58]
		23.45%	DPPH	[58]
	Methanolic extract	87.81%	DPPH	[58]
		71.49%	Hydrogen peroxide scavenging activity	[58]
		97.7 mg Trolox equivalents (TE)/g DM	ORAC	[57]
	Petroleum ether fraction	21.02% inhibition at 600 µg/mL	DPPH	[54]
*H. stipulacea*	Ethanolic extract	46.289 mg gallic acid/g	FRAP	[55]
		67.41%	DPPH	[55]
*H. stipulacea (old leaf extract)*	EtOH/H_2_O (3:1)	85% inhibition at 100 µg/mL	DPPH	[41]
*H. stipulacea (young leaf extract)*		45% inhibition at 100 µg/mL	DPPH	[41]
*S. filiforme*	MeOH/H_2_O (1:1)	IC_50_: 0.8 mg/mL	DPPH	[59]
*S. isoetifolium*	Acetone extract	45.69%	DPPH	[58]
		49.24%	NO scavenging activity	[58]
	Aqueous fraction	16.2% inhibition at 600 µg/mL	DPPH	[54]
	Aqueous methanol (1:4)	51.56%	DPPH	[52]
		48.42%	Hydroxyl radical scavenging activity	[52]
	Butanol fraction	6.2% inhibition at 600 µg/mL	DPPH	[54]
	Ethanolic extract	26.557 mg gallic acid/g	FRAP	[55]
		23.68%	DPPH	[55]
	Ethyl acetate	50% inhibition at 96.34 ppm	DPPH	[53]
	Ethyl acetate fraction	6.36% inhibition at 600 µg/mL	DPPH	[54]
	Hexane extract	15.19%	DPPH	[58]
		51.49%	NO Scavenging Activity	[58]
	Methanolic extract	83.03%	DPPH	[58]
		50% inhibition at 520.91 ppm	DPPH	[53]
		5.39 mgTE/g	DPPH	[60]
		9.56 mgTE/g	ABTS	[60]
		18.66 mgTE/g	CUPRAC	[60]
		9.53 mgTE/g	FRAP	[60]
		0.33 mmolTE/g	PHPD	[60]
		9.17 mgEDTAE/g	Chelating ability	[60]
	Petroleum ether fraction	10.2% inhibition at 600 µg/mL	DPPH	[54]
*T. ciliatum*	Catechins	50% inhibition at 3.82 mM	DPPH	[17]
	Methanolic extract	71% inhibition at 1 mg/mL	DPPH	[17]
*T. hemprichii*	Aqueous fraction	26.6% inhibition at 600 µg/mL	DPPH	[54]
	Aqueous methanol (1:4)	38.62%	DPPH	[52]
		35.25%	Hydroxyl radical scavenging activity	[52]
	Butanol fraction	84.9% inhibition at 600 µg/mL	DPPH	[54]
	Ethanolic extract	27.979 mg gallic acid/g	FRAP	[55]
		61.64%	DPPH	[55]
	Ethyl acetate extract	IC_50_: 25.98 µg/mL	DPPH	[61]
		50% inhibition at 250.72 ppm	DPPH	[53]
	Ethyl acetate fraction	94.34% inhibition at 600 µg/mL	DPPH	[54]
	Hexane extract	IC_50_: 139.5 µg/mL	DPPH	[61]
	Methanolic extract	50% inhibition at 123.72 ppm	DPPH	[53]
	Petroleum ether fraction	42.67% inhibition at 600 µg/mL	DPPH	[54]
*T. testudinum*	MeOH/H_2_O (1:1)	IC_50_: 0.8 mg/mL	DPPH	[59]

### 3.3. Anti-Inflammatory Effects

Inflammation is a complex physiological response to various harmful stimuli characterized by the recruitment and activation of immune cells (innate and adaptive immunity), which rapidly manage the resolution and healing of damaged tissues [62]. An uncontrolled immune response can make inflammation a pathological condition. Inflammation leads to a reduction in the pain threshold, inducing pathological hypersensitivity and resulting in persistent pain [63]. The failure of a rapid resolution can evolve into chronic inflammation, which could determine the onset of inflammatory diseases or the development of cancer [64]. Macrophage cells play a significant role in immune responses and inflammatory processes, covering a wide variety of functions, such as the activation of inflammation and regulation of tissue repair [65]. Lipopolysaccharide (LPS) is one of the most widely used pro-inflammatory stimuli that can activate macrophages and trigger the inflammatory response [66]. The ethanolic extract of *P. oceanica* decreased the LPS-induced high levels of COX2, thus exhibiting an anti-inflammatory role associated with antioxidant effects [67]. This extract also exhibited a strong ability to inhibit oxidative stress by affecting the production of both ROS and NO radicals, as well as by reducing iNOS and COX-2 levels [67]. In addition, its anti-inflammatory role via inhibiting the NF-κB-signaling pathway through modulation of ERK1/2 and Akt intracellular cascades was evidenced [67].

Palmitoleic acid (**36**) isolated from *S. filiforme* and *T. testudinum* had potent anti-inflammatory activity by inhibiting the LPS-induced release of TNF-α, IL-1β, IL-6, MIP-3α, and l-selectin [68]. Compound **21** revealed powerful anti-inflammatory properties through MAPK and NF-κB-signaling pathways [69]. Stearic acid (**42**) isolated from *S. filiforme* and *T. testudinum* mitigated the inflammatory response by inhibiting neutrophil migration, thereby reducing TNF-α and IL-1β [70]. Compound **24** acted as an anti-inflammatory agent by suppressing the expression of pro-inflammatory cytokines [71]. The administration of *P. oceanica* extract at 10–100 mg/kg in a dose-dependent manner increased the pain threshold; the higher dose was significantly effective between 15 and 45 min after treatment, completely blocking carrageenan-induced hypersensitivity [63]. The underlying mechanism is the reduction in the TNF-α and IL-1β levels that play an important role in inflammation pathways [63]. On the other hand, the methanolic extract of *H. ovalis* exhibited 50% inhibition of the proliferation of peripheral blood mononuclear cells (PBMCs) at 78.72 μg/mL [39]. Compound **14** isolated from *T. ciliatum* at 20 mg/kg has been found to possess significant anti-inflammatory activity according to the carrageenan-induced rat paw edema test [19]. The anti-inflammation pathway of the hydroethanolic extract of *P. oceanica* is shown in Figure 4 [72,73].

### 3.4. Antibacterial Activity

Infectious diseases remain a major cause of death due to multidrug resistance. The emergence and spread of antibiotic-resistant pathogens are a great concern to the global health community. Annual deaths due to antimicrobial resistance in the world are projected to reach up to 10 million by the year 2050 [74]. Increasing pathogen resistance rates and the ineffectiveness of antibiotics have spurred research on other options. The effective treatment of a disease entails the development of new pharmaceuticals or potential sources of novel drugs. Commonly used medicinal plants could be an excellent source of drugs to resolve this problem [75]. According to the World Health Organization (WHO), medicinal plants could be the best source for obtaining a variety of drugs, as they produce several bioactive compounds with known therapeutic properties. Many plant extracts exhibit a good antibacterial activity towards different tested bacterial isolates, as indicated by their MIC values [76]. Antimicrobial agents are essential to reducing the global burden of infectious diseases. They act through several mechanisms, including damaging bacterial wall permeability, microsomes, and lysosomes; acting as DNA-intercalating agents; and inhibiting the reverse transcriptase and topoisomerase enzymes [77]. The biological activity of alkaloid compounds caused the presence of nitrogenous groups that react with amino acids and change their arrangement [77]. This process destroys the genetic balance in DNA so that bacterial DNA is damaged. DNA damage in the bacterial cell nucleus prevents the bacteria’s metabolism, leading to cell lysis [77].

Seagrasses produce antimicrobial compounds that may reduce or control microbial growth, and many reports have described their antibacterial activity [78] (Figure 5). 

4-Hydroxybenzaldehyde (**27**) isolated from chloroform fraction of *T. testudinum* (1.55%) exhibited synergism with amphenicol antibiotic to treat MDR infection [79]. Isoscutellarein (**28**) isolated from *T. hemprichii* showed antibacterial activity against *B. subtilis*, *S. aureus*, *E. coli*, and *P. aeruginosa*, with MIC 4.3 μg/mL, 4.2 μg/mL, 5 μg/mL, and 3.2 μg/mL, respectively [80]. Remarkably, compound **30**, the 8-hydroxy flavone O-xyloside sulfate derivative, exhibited more potent activity against *B. subtilis* (2.5 μg/mL) and *P. aeruginosa* (1.5 μg/mL) than compounds **28** and **29** at the same concentration [80]. Moreover, 3-hydroxybutyric acid (**29**) isolated from the chloroform fraction of *S. filiforme* (0.44%) inhibited the growth of *S. aureus* and *K. pneumonia*. The underlying mechanisms were revealed, including disruption of biofilm and the bacterial wall/membrane, leakage of the intracellular content, inhibition of protein activity, and changes in the transmembrane potential [81]. Pentadecanoic acid (**32**) isolated from the chloroform fractions of *S. filiforme* (0.47%) and *T. testudinum* (1.34%) inhibited the growth of *K. pneumoniae* polymicrobial biofilm [82]. Lauric acid (**33**) isolated from chloroform fractions of *S. filiforme* (3.16%) and *T. testudinum* (0.72%) demonstrated a 15-mm zone of inhibition on *S. aureus* and *S. pneumoniae* [83]. Tricosane (**34**) isolated from chloroform fractions of *S. filiforme* (1.93%) was active against *P. fluorescens* SHL7, *E. coli*, *B. subtilis*, and *S. cerevisiae* at a concentration of 20 mg/mL, with inhibition zones ranging from 8.03 to 15.97 mm [84]. Octanoic acid (**35**) isolated from chloroform fractions of *S. filiforme* (1.53%) and *T. testudinum* (0.2%) can inactivate *E. coli* by damaging the cell membrane and inhibiting the metabolic activity [85]. Palmitoleic acid (**36**) isolated from chloroform fractions of *S. filiforme* (6.47%) and *T. testudinum* (2.81%) showed an appreciable killing effect against *H. pylori*, *Streptococcus* sp., and *N. gonorrhoeae* [86]. Linoleic acid (**37**) isolated from aqueous-methanolic extracts of *H. pinifolia* (5.76%), *E. acoroides* (2.6%), *C. serrulata* (12.28%), and *C. rotundata* (17.67%) exhibited strong antibacterial activity against *L. monocytogenes* at 10–20 µg/mL [86]. It also has been reported that compound **37** damaged *S. aureus* cell membranes by inducing a marked increase in membrane permeability [86].

Oleic acid (**38**) isolated from methanolic extracts of *H. ovalis* (27.01%) aggregates around individual cells of group *A. streptococci* and interacts with the bacterial cell membrane [86]. Arachidonic acid (**39**) isolated from chloroform fractions of *T. testudinum* (1.34%) induced the significant membrane disruption of *N. gonorrhoeae* at a concentration of 10 µM [86]. Myristic acid (**40**) isolated from methanolic extracts of *H. ovalis* (6.12%) exhibited antimicrobial activity against *B. megaterium*, *Pneumococci*, *Streptococcus group A*, *Micrococcus* sp., *Corynebacterium* sp., and *N. asteroids*, with MIC values of 0.15 mM, 0.218 mM, 0.547 mM, 0.547 mM, 0.437 mM, and 0.547 mM, respectively [86]. Palmitic acid (**41**) isolated from ethanolic extracts of *C. serrulata* (14.11%), chloroform fractions of *S. filiforme* (39.18%), and *T. testudinum* (50.21%) exhibited antimicrobial activity against *B. megaterium* and *Pneumococci*, with MIC 0.3 mM and 0.48 mM [86]. Stearic acid (**42**) isolated from chloroform fractions of *S. filiforme* (2.19%) and *T. testudinum* (3.14%) showed antimicrobial activity against *B. megaterium*, with MIC 0.4 mM [86]. Linolenic acid (**43**) isolated from aqueous methanolic extracts of *H. pinifolia* (22.83%) revealed antimicrobial activity against *B. megaterium* and *Pneumococci* with MIC 0.02 mM and 0.179 mM [86]. Myristioleic acid (**44**) isolated from chloroform fractions of *T. testudinum* (0.97%) showed potent antibacterial activity against *B. larvae* [86]. Phytol (**45**) isolated from aqueous-methanolic extracts of *T. hemprichii* (13.49%), *E. acoroides* (16.6%), *C. serrulata* (1.94%), and *C. rotundata* (7.23%) inhibited the growth of *E. coli* and *P. aeruginosa*, with MIC 62.5 μg/mL and 19 μg/mL, respectively [70]. Compound **16** exhibited antibacterial activity against *S. typhii*, with MIC 6.2 μg/mL and MBC 12.5 μg/mL [87].

Extracts of *C. rotundata*, *C. serrulata*, *E. acoroides*, *H. pinifolia*, *H. ovalis*, *H. uninervis*, *H. stipulacea*, *S. filiforme*, *S. isoetifolium*, and *T. hemprichii* were found to inhibit the growth of test bacteria (Table 4). Among the tested solvents, the methanolic extracts of *C. serrulate* and *S. isoetifolium* showed better inhibitory activity than other solvents against the biofilm bacteria, and the MIC was 1.0 μg/mL [88]. In another study, the methanol extract of seagrass *H. ovalis* collected from Chunnambar estuary, Pondicherry coastal line, exhibited antibacterial activity against Gram-positive *B. cereus* and Gram-negative pathogens such as *V. parahaemolyticus, V. fischeri, V. anguillarum, V. vulnificus*, and *A. baumannii* (Table 4). Similarly, extracts from *C. rotundata* Ehrenberg and Hemprich ex Ascherson (Cymodoceaceae) were effective against *Bacillus* species [39]. Hexane and methanolic extracts of *E. acoroides* are thought to be capable of interfering with peptidoglycans and damaging bacteria cell membranes, thus inhibiting the growth of pathogenic bacteria [89]. Moreover, flavonoid compounds isolated from various seagrass species act as antibacterial agents by reducing the permeability of cell walls [89]. Pheophytin isolated from *S. isoetifolium* inhibits the growth of *S. typhii* by binding to the umuC proteins of the bacteria and stalling cell cycle progression instantaneously [87].

**Table 4:** Antibacterial activity of seagrass extracts.

Species	Extract Type/Active Compound	Bacteria	Inhibition	References
*C. nodosa*	Sulfated polysaccharide	*E. coli*	MIC: 25 mg/mL 20 mm zone inhibition	[51]
		*L. monocytogene*	MIC: 25 mg/mL 18 mm zone inhibition	[51]
		*S. enterica*	MIC: 6.25 mg/mL 21.5 mm zone inhibition	[51]
		*B. subtilis*	MIC: 6.25 mg/mL 18.6 mm zone inhibition	[51]
		*B. amyloliquefaciens*	MIC: 50 mg/mL 17 mm zone inhibition	[51]
		*S. aureus*	MIC: 25 mg/mL 23 mm zone inhibition	[51]
		*M. luteus*	MIC: 6.25 mg/mL 24 mm zone inhibition	[51]
*C. rotundata*	Aqueous methanol (1:4)	*S. dysenteriae*	MIC: 68 μg/mL	[90]
		*S. boydii*	MIC: 68 μg/mL	[90]
		*S. paratyphi*	MIC: 34 μg/mL	[90]
		Urinary tract infection (UTI) bacteria	MIC: 10 μg/mL MBC: 50 μg/mL	[91]
		*E. coli*	10 mm zone inhibition	[91]
		*P. mirabilis*	12 mm zone inhibition	[91]
		*S. saprophyticus*	11.6 mm zone inhibition	[91]
		*K. pneumonia*	11.3 mm zone inhibition	[91]
		*P. aeruginosa*	12.3 mm zone inhibition	[91]
		*E. aerogenes*	9.7 mm zone inhibition	[91]
		*Serratia* sp.	10 mm zone inhibition	[91]
	Aqueous methanolic extracts	*S. dysenteriae*	MBC: 68 μg/mL	[90]
		*S. boydii*	MBC: 34 μg/mL	[90]
	Butanolic extract	*S. aureus*	6 mm zone inhibition	[78]
	Ethanolic extract	*Shigella*	7 mm zone inhibition	[78]
		*P. fluorescens*	7 mm zone inhibition	[78]
	Methanolic extract	*S. aureus*	17 mm zone inhibition	[92]
		*S. faecalis*	13 mm zone inhibition	[92]
		*S. enteric*	8 mm zone inhibition	[92]
		*B. subtilis*	14 mm zone inhibition	[92]
		*E. coli*	15 mm zone inhibition	[92]
		*S. boydii*	8 mm zone inhibition	[92]
		*V. cholera*	8 mm zone inhibition	[92]
*C. serrulata*	Acetone extract	*P. aeruginosa*	MIC: 25 μg/mL	[88]
		*H. aquamarina*	MIC: 50 μg/mL	[88]
		*P. agglomerans*	MIC: 25 μg/mL	[88]
		*S. marcescens*	MIC: 50 μg/mL	[88]
		*S. liquefaciens*	MIC: 10 μg/mL	[88]
		*V. fischeri*	MIC: 25 μg/mL	[88]
		*V. parahaemolyticus*	MIC: 50 μg/mL	[88]
		*S. flexneri*	MIC: 50 μg/mL	[88]
	Aqueous methanol (1:4)	*S. dysenteriae*	MIC: 130 μg/mL	[90]
		*S. paratyphi*	MIC: 131 μg/mL	[90]
		Urinary tract infection (UTI) bacteria	MIC: 100 μg/mL	[12]
		*S. saprophyticus*	6 mm zone inhibition	[91]
		*P. aeruginosa*	6.3 mm zone inhibition	[91]
	Aqueous methanolic extracts	*S. dysenteriae*	MBC: 130 μg/mL	[90]
		*S. paratyphi*	MBC: 130 μg/mL	[90]
	Chloroform extract	*Corynebacterium*	MIC: 850 μg/mL 7 mm zone inhibition	[93]
		*E. coli*	MIC: 90 μg/mL 8.66 mm zone inhibition	[93]
	Dichloromethane extract	*P. aeruginosa*	MIC: 10 μg/mL	[88]
		*H. aquamarina*	MIC: 25 μg/mL	[88]
		*P. agglomerans*	MIC: 25 μg/mL	[88]
		*S. marcescens*	MIC: 10 μg/mL	[88]
		*S. liquefaciens*	MIC: 50 μg/mL	[88]
		*V. fischeri*	MIC: 10 μg/mL	[88]
		*V. parahaemolyticus*	MIC: 25 μg/mL	[88]
		*S. flexneri*	MIC: 25 μg/mL	[88]
		*A. hydrophila*	MIC: 10 μg/mL	[88]
	Ethanolic extract	*E. coli*	MIC: 90 μg/mL 7.33 mm zone inhibition	[93]
	Ethyl acetate extract	*Corynebacterium*	MIC: 875 μg/mL 8 mm zone inhibition	[93]
		*E. coli*	MIC: 75 μg/mL 9 mm zone inhibition	[93]
	Methanolic extract	*P. aeruginosa*	MIC: 10 μg/mL	[88]
		*H. aquamarina*	MIC: 1 μg/mL	[88]
		*V. alginolyticus*	MIC: 10 μg/mL	[88]
		*P. agglomerans*	MIC: 1 μg/mL	[88]
		*S. marcescens*	MIC: 1 μg/mL	[88]
		*S. liquefaciens*	MIC: 10 μg/mL	[88]
		*V. fischeri*	MIC: 1 μg/mL	[88]
		*V. parahaemolyticus*	MIC: 10 μg/mL	[88]
		*S. flexneri*	MIC: 25 μg/mL	[88]
		*A. hydrophila*	MIC: 1 μg/mL	[88]
*E. acoroides*	Aqueous methanol (1:4)	Urinary tract infection (UTI) bacteria	MIC: 25 μg/mL MBC: 100 μg/mL	[91]
		*P. mirabilis*	9.3 mm zone inhibition	[91]
		*K. pneumonia*	8.3 mm zone inhibition	[91]
		*P. aeruginosa*	9.3 mm zone inhibition	[91]
		*E. aerogenes*	8.7 mm zone inhibition	[91]
		*Serratia* sp.	6.3 mm zone inhibition	[91]
	Ethanolic extract	*E. coli*	MIC: 250 μg/mL	[94]
		*S. aureus*	MIC: 62.5 μg/mL	[94]
		*B. subtilis*	MIC: 250 μg/mL	[94]
	Ethanolic extract (leaves)	*S. aureus*	8.37 mm at 400 ppm	[95]
	Ethanolic extract (roots)	*S. aureus*	8.63 mm at 400 ppm	[95]
	Ethyl acetate	*E. coli*	MIC: 31.25 μg/mL	[94]
		*S. aureus*	MIC: 31.25 μg/mL	[94]
		*B. subtilis*	MIC: 62.5 μg/mL	[94]
	Hexane extract	*S. aureus*	MIC: 15.625 μg/mL 5.6 mm zone inhibition at 1000 ppm 5.2 mm zone inhibition at 500 ppm	[89,94]
			19 mm zone inhibition	[96]
		*E. coli*	MIC: 31.25 μg/mL	[94]
		*B. subtilis*	MIC: 250 μg/mL	[94]
	Methanolic extract	*S. aureus*	5.9 mm zone inhibition at 1000 ppm	[89]
			5.2 mm zone inhibition at 500 ppm	[89]
		*E. coli*	60.86% inhibition at 10 mg/mL	[57]
*H. ovalis*	Chloroform extract	*Corynebacterium*	MIC: 65 μg/mL 11.66 mm zone inhibition	[93]
		*E. coli*	MIC: 225 μg/mL 7.6 mm zone inhibition	[93]
	Ethanolic extract	*E. coli*	MIC: 90 μg/mL 7 mm zone inhibition	[93]
	Ethyl acetate extract	*Corynebacterium*	MIC: 65 μg/mL 13 mm zone inhibition	[93]
		*E. femelis*	MIC: 85 μg/mL 7.66 mm zone inhibition	[93]
		*E. coli*	MIC: 90 μg/mL 7.33 mm zone inhibition	[93]
	Hexane extract	*Corynebacterium*	MIC: 50 μg/mL 14.66 mm zone inhibition	[93]
		*E. coli*	MIC: 435 μg/mL 7 mm zone inhibition	[93]
	Methanolic extract	*B. cereus*	MIC: 50 μg/mL 17.16 mm zone inhibition at 200 μg/mL	[39]
		*A. baumannii*	MIC: 75 μg/mL 13.83 mm zone inhibition at 200 μg/mL	[39]
		*V. vulnificus*	MIC: 100 μg/mL 10.36 mm zone inhibition at 200 μg/mL	[39]
		*V. parahaemolyticus*	MIC: 75 μg/mL 10.16 mm zone inhibition at 200 μg/mL	[39]
		*V. anguillarum*	MIC: 75 μg/mL 10.16 mm zone inhibition at 200 μg/mL	[39]
		*V. fischeri*	MIC: 75 μg/mL 10 mm zone inhibition at 200 μg/mL	[39]
		*E. coli*	MIC: 75 μg/mL 8.53 mm zone inhibition at 200 μg/mL	[39]
		*M. luteus*	MIC: 50 μg/mL	[39]
*H. pinifolia*	Aqueous methanol (1:4)	Urinary tract infection (UTI) bacteria	MIC: 1 μg/mL MBC: 25 μg/mL	[91]
		*E. coli*	12.3 mm zone inhibition	[91]
		*P. mirabilis*	13.7 mm zone inhibition	[91]
		*S. saprophyticus*	10.7 mm zone inhibition	[91]
		*K. pneumonia*	11.7 mm zone inhibition	[91]
		*P. aeruginosa*	10.3 mm zone inhibition	[91]
		*E. aerogenes*	14.3 mm zone inhibition	[91]
		*Serratia* sp.	11.3 mm zone inhibition	[91]
		*S. dysenteriae*	MIC: 34 μg/mL	[90]
		*S. paratyphi*	MIC: 509 μg/mL	[90]
		*S. boydii*	MIC: 510 μg/mL	[90]
	Aqueous methanolic extracts	*S. dysenteriae*	MBC: 34 μg/mL	[90]
		*S. paratyphi*	MBC: 510 μg/mL	[90]
		*S. boydii*	MBC: 510 μg/mL	[90]
	Chloroform extract	*Corynebacterium*	MIC: 55 μg/mL 13.66 mm zone inhibition	[93]
		*E. coli*	MIC: 90 μg/mL 8.33 mm zone inhibition	[93]
	Ethanolic extract	*E. coli*	MIC: 80 μg/mL 8 mm zone inhibition	[93]
	Ethyl acetate extract	*Corynebacterium*	MIC: 35 μg/mL 11 mm zone inhibition	[93]
		*E. coli*	MIC: 70 μg/mL 9 mm zone inhibition	[93]
	Hexane extract	*Corynebacterium*	MIC: 50 μg/mL 14.33 mm zone inhibition	[93]
*H. stipulaceae*	Aqueous extract	*B. subtilis*	15 mm zone inhibition	[97]
*H. uninervis*	Chloroform extract	*B. subtilis*	17 mm zone inhibition	[75]
		*MRSA*	18.33 mm zone inhibition	[75]
		*M. luteus*	15/67 mm zone inhibition	[75]
		*S. aureus*	13.67 mm zone inhibition	[75]
		*E. coli*	16.33 mm zone inhibition	[75]
		*K. pneumoniae*	17.67 mm zone inhibition	[75]
		*P. aeruginosa*	18.33 mm zone inhibition	[75]
	Distilled water	*P. aeruginosa*	14.67 mm zone inhibition	[75]
	Ethanolic extract	*B. subtilis*	24.67 mm zone inhibition	[75]
		*MRSA*	20 mm zone inhibition	[75]
		*M. luteus*	17.33 mm zone inhibition	[75]
		*S. aureus*	15.67 mm zone inhibition	[75]
		*E. coli*	17.33 mm zone inhibition	[75]
		*K. pneumoniae*	18.67 mm zone inhibition	[75]
		*P. aeruginosa*	33.33 mm zone inhibition	[75]
	Ethyl acetate extract	*B. subtilis*	15.67 mm zone inhibition	[75]
		*MRSA*	16.67 mm zone inhibition	[75]
		*M. luteus*	15 mm zone inhibition	[75]
		*S. aureus*	12.67 mm zone inhibition	[75]
		*E. coli*	15.33 mm zone inhibition	[75]
		*K. pneumoniae*	16 mm zone inhibition	[75]
		*P. aeruginosa*	16.67 mm zone inhibition	[75]
	Petroleum ether extract	*B. subtilis*	15.67 mm zone inhibition	[75]
		*MRSA*	16 mm zone inhibition	[75]
		*M. luteus*	14.33 mm zone inhibition	[75]
		*S. aureus*	11.67 mm zone inhibition	[75]
		*E. coli*	14.33 mm zone inhibition	[75]
		*K. pneumoniae*	14.47 mm zone inhibition	[75]
		*P. aeruginosa*	15.67 mm zone inhibition	[75]
*S. filiforme*	Chloroform fraction	*S. aureus*	MIC: 0.7 mg/mL	[34]
		*E. coli*	MIC: 0.7 mg/mL	[34]
		*C. albicans*	MIC: 1.5 mg/mL	[34]
		*P. aeruginosa*	MIC: 1.5 mg/mL	[34]
		*S. typhii*	MIC: 0.7 mg/mL	[34]
	Ethanolic extract	*S. aureus*	MIC: 47.7 mg/mL	[34]
		*E. coli*	MIC: 38.1 mg/mL	[34]
		*C. albicans*	MIC: 190.5 mg/mL	[34]
*S. isoetifolium*	Acetone extract	*P. aeruginosa*	MIC: 25 μg/mL	[88]
		*H. aquamarina*	MIC: 25 μg/mL	[88]
		*V. alginolyticus*	MIC: 50 μg/mL	[88]
		*S. marcescens*	MIC: 25 μg/mL	[88]
		*S. liquefaciens*	MIC: 50 μg/mL	[88]
		*V. parahaemolyticus*	MIC: 50 μg/mL	[88]
		*S. flexneri*	MIC: 25 μg/mL	[88]
		*A. hydrophila*	MIC: 50 μg/mL	[88]
	Aqueous methanol (1:4)	Urinary tract infection (UTI) bacteria	MIC: 50 μg/mL MBC: 100 μg/mL	[91]
		*P. mirabilis*	8.7 mm zone inhibition	[91]
		*S. saprophyticus*	8.3 mm zone inhibition	[91]
		*E. aerogenes*	7 mm zone inhibition	[91]
	Ethyl acetate extract	*K. pneumoniae*	14 mm zone inhibition at 100 μg/mL	[87]
		*E. coli*	13 mm zone inhibition at 100 μg/mL	[87]
		*S. typhii*	11 mm zone inhibition at 100 μg/mL	[87]
	Dichlorometahane extract	*A. hydrophila*	MIC: 10 μg/mL	[88]
		*P. aeruginosa*	MIC: 10 μg/mL	[88]
		*H. aquamarina*	MIC: 10 μg/mL	[88]
		*V. alginolyticus*	MIC: 25 μg/mL	[88]
		*P. agglomerans*	MIC: 25 μg/mL	[88]
		*S. marcescens*	MIC: 10 μg/mL	[88]
		*S. liquefaciens*	MIC: 50 μg/mL	[88]
		*V. fischeri*	MIC: 10 μg/mL	[88]
		*S. flexneri*	MIC: 25 μg/mL	[88]
	Methanolic extract	*P. aeruginosa*	MIC: 1 μg/mL	[88]
		*H. aquamarina*	MIC: 10 μg/mL	[88]
		*V. alginolyticus*	MIC: 25 μg/mL	[88]
		*P. agglomerans*	MIC: 1 μg/mL	[88]
		*S. marcescens*	MIC: 10 μg/mL	[88]
		*S. liquefaciens*	MIC: 10 μg/mL	[88]
		*V. fischeri*	MIC: 10 μg/mL	[88]
		*V. parahaemolyticus*	MIC: 25 μg/mL	[88]
		*S. flexneri*	MIC: 1 μg/mL	[88]
		*A. hydrophila*	MIC: 10 μg/mL	[88]
		*S. aureus*	15 mm zone inhibition	[92]
		*S. faecalis*	10 mm zone inhibition	[92]
		*S. enteric*	6 mm zone inhibition	[92]
		*B. subtilis*	10 mm zone inhibition	[92]
		*E. coli*	8 mm zone inhibition	[92]
		*V. cholera*	6 mm zone inhibition	[92]
*T. hemprichii*	Aqueous methanol (1:4)	Urinary tract infection (UTI) bacteria	MIC: 25 μg/mL MBC: 50 μg/mL	[91]
		*E. coli*	9.3 mm zone inhibition	[91]
		*P. mirabilis*	10.3 mm zone inhibition	[91]
		*S. saprophyticus*	9.3 mm zone inhibition	[91]
		*K. pneumonia*	11.3 mm zone inhibition	[91]
		*P. aeruginosa*	10.6 mm zone inhibition	[91]
		*E. aerogenes*	9.3 mm zone inhibition	[91]
		*Serratia* sp.	8.7 mm zone inhibition	[91]
	Ethanolic extract	*E. coli*	MIC: 500 μg/mL	[94]
		*S. aureus*	MIC: 125 μg/mL	[94]
		*B. subtilis*	MIC: 500 μg/mL	[94]
	Ethyl acetate extract	*E. coli*	MIC: 125 μg/mL	[94]
		*S. aureus*	MIC: 250 μg/mL	[94]
		*B. subtilis*	MIC: 125 μg/mL	[94]
	Hexane extract	*E. coli*	MIC: 62.5 μg/mL	[94]
		*S. aureus*	MIC: 62.5 μg/mL	[94]
		*B. subtilis*	MIC: 125 μg/mL	[94]

### 3.5. Antifungal Treatments

Fungal infections are caused by eukaryotic organisms, and it is more difficult to ascertain their presence and apply the appropriate therapeutic treatment compared to bacterial infections [98]. Over the last decades, control of pathogenic fungi has become a critical challenge due to an increase in the incidence of fungal infections and the emergence of antifungal-resistant strains. The onset and severity of the fungal infections depend on the inoculum charge, the host’s immunological state, and resistance [99]. Patients who receive immunosuppressive agents such as cancer therapy and immunocompromised patients can be vulnerable to fungal infections [100]. Fungal diseases kill more than 1.5 million and affect over a billion people in the world. Since 2013, the Leading International Fungal Education (LIFE) portal estimated the burden of serious fungal infections for over 5.7 billion people (>80% of the world’s population) [101].

Fungi cell walls are considered the prime target for selectively toxic antifungal agents because of their chitin structure, which is absent in human cells [102]. Fungal infection treatments are very limited when compared to bacterial infections. The rise in fungal infection incidence has exacerbated the urgency for new antifungal agents, as many available drugs have several side effects, are ineffective against new or re-emerging fungal strains, and lead to the rapid development of resistance [103]. Ideally, new antifungals should combine major aspects such as sustainability, high efficacy, limited toxicity, and low cost of production. Previous studies showed that seagrasses produced secondary metabolites with a defensive role against marine pathogens. Isoscutellarein (**28**) and its glycosylated derivatives (**29**,**30**) isolated from *T. hemprichii* showed antifungal activity against *A. niger* and *C. albicans* with MIC values between 5 and 8 μg/mL [80]. Flavone glycosides isolated from *T. testudinum* were reported to inhibit the growth of the thraustochytrid (zoosporic fungus) *Schizochytrium aggregatum* [104]. The antifungal activities of different seagrasses are summarized in Table 5.

**Table 5:** Antifungal activities of seagrasses.

Species	Extract/Active Compounds	Fungus	Activity	References
*C. nodosa*	Sulfated polysaccharide	*A. niger*	Zone of inhibition: 15 mm MIC: 6.25 mg/mL	[51]
		*F. oxysporum*	Zone of inhibition: 14.3 mm MIC: 12.5 mg/mL	[51]
		*C. albicans*	Zone of inhibition: 18 mm MIC: 12.5 mg/mL	[51]
		*C. neoformans (flucytosine sensitive)*	MIC: 16 μg/mL MBC > 200 μg/mL	[105]
		*C. neoformans (flucytosine resistant)*	MIC: 8 μg/mL MBC: 128 μg/mL	[105]
		*M. gypseum*	MIC: 2 μg/mL MBC: 16 μg/mL	[105]
*C. rotundata*	Methanolic extract	*A. niger*	Zone of inhibition: 15 mm antifungal activity index: 83%	[92]
		*A. fumigates*	Zone of inhibition: 8 mm antifungal activity index: 67%	[92]
		*Fusarium*	Zone of inhibition: 10 mm antifungal activity index: 10%	[92]
*E. acoroides*	Methanolic extract	*C. albicans*	Reduces fungal coverage up to 73.89 ± 1.01% at 0.01 mg/L	[57]
*H. ovalis*	Methanolic extract	*C. albicans*	Reduces fungal coverage up to 68.37 ± 2.49% at 1 mg/L	[57]
*H. stipulaceae*	Aqueous extract	*A. niger*	Zone of inhibition: 20 mm	[97]
		*C. albicans*	Zone of inhibition: 15 mm	[97]
*S. isoetifolium*	Methanolic extract	*A. niger*	Zone of inhibition: 12 mm antifungal activity index: 67%	[92]
		*A. fumigates*	Zone of inhibition: 6 mm antifungal activity index: 50%	[92]
		*Fusarium*	Zone of inhibition: 8 mm antifungal activity index: 8%	[92]
*T. hemprichii*	Hexane/ethanol (3:1)	*F. acuminatum*	Zone of inhibition: 2.5 mm	[106]
		*A. niger*	Zone of inhibition: 1.7 mm	[106]
		*P. expansum*	Zone of inhibition: 2.1 mm	[106]
		*A. terrus*	Zone of inhibition: 3.2 mm	[106]
		*A. fumigatus*	Zone of inhibition: 1.5 mm	[106]

### 3.6. Antiviral Activity

Infectious viral diseases remain a global problem. Viruses have been resistant to therapy or prophylaxis longer than any form of life because they completely depend on the cells they infect for their multiplication and survival. Currently, there are only a few drugs available to cure viral diseases, including acyclovir, the known antiherpetic drug modeled on a natural product parent. A number of life-threatening viruses, including human immunodeficiency virus (HIV), adenovirus (ADV), hepatitis virus (HAV, HBV, and HCV), herpes simplex virus (HSV), and influenza virus, have affected human health for a long time [107]. Many research efforts have been devoted to the discovery of new antiviral natural products to combat viruses that have devastating effects on humans, animals, insects, crop plants, fungi, and bacteria. Many recent studies have revealed the antiviral activity of various seagrasses; these are summarized in Table 6.

Thalassodendrone (**46**), a 6-O-rhamnosyl-glucopyranosyl asebogenin, and asebotin (**7**) isolated from *T. ciliatum* reduced influenza A virus toxicity with cytotoxic concentration (CC_50_) 3.14 μg/mL and 3.36 μg/mL, respectively [108]. Thalassiolin D (**47**) isolated from methanolic extract of *T. hemprichii* inhibited HCV with IC_50_ 16 μM [109]. Compound **47** showed antiviral activity against HCV through the inhibition of the HCV NS3-NS4A protease [109]. Phthalic acid (**48**) isolated from chloroform/methanolic fractions of *C. serrulata* (4.35%) exhibited significant activity against HIV protease through in silico studies [110]. Erucic acid (**49**) isolated from chloroform/methanolic fractions of *C. serrulata* (15.68%) suppressed influenza A virus replication through the modulation of the NF-κB and p38 MAPK pathway [111]. Octadecanoic acid (**50**) isolated from aqueous-methanolic extracts of *E. acoroides* (8.18%), hydroethanolic extracts of *S. isoetifolium* (29.99%), and methanolic extracts of *H. ovalis* (10.42%) exhibited HIV-1 protease inhibition through bind to 3NU3 protein with a binding affinity of −8.5 kcal/mol [110]. Hexadecanoic acid (**51**) isolated from methanolic extract of *H. ovalis* (21.63%), as well as aqueous-methanolic extracts of *E. acoroides* (24.59%), *T. hemprichii* (32.86%), *H. pinifolia* (14.75%), *S. isoetifolium* (42.88%), *C. serrulata* (35.74%), and *C. rotundata* (55.55%), exhibited HIV-1 protease inhibitor by bonding to 3NU3 proteins with a binding affinity of −7.2 kcal/mol [110]. β-sitosterol (**52**) isolated from chloroform fractions of *S. filiforme* (0.14%) and *T. testudinum* (0.83%) can be used to restrict SARS-CoV-2 invasion into the host cell through angiotensin-converting enzyme-2 (ACE-2) by inhibiting spike glycoprotein [112]. Stigmasterol (**53**) isolated from ethanolic extracts of *C. serrulata* (19.42%), chloroform fractions of the ethanolic extract of *S. filiforme* (0.13%), and hydroethanolic extracts of *T. testudinum* (0.72%) hindered interleukin-6 and interferon-gamma secretion induced by HSV-1 infection in Neuro-2a cells [111]. Compounds **1**, **7**, and **8** at 1 mg/mL concentration have been reported to inhibit HSV-1 growth by 70%, 96.6%, and 53% respectively [17]. Moreover, compound **7** inhibited the influenza A virus, with IC_50_ 2 μg/mL [108] (Figure 6).

Methanolic extract and several compounds isolated from seagrasses indicate a significant inhibition in numerous viral types, including HAV, HSV-1, HSV-2, ADV-3, and influenza A [17]. Polyphenol complex from the Zosteraceae family act as antiviral by directly inactivating the tick-borne encephalitis (TBE) virus and inhibiting the virus’s replication at the early stage, thus indicating a reduction in virus titer [113]. The methanolic extract of *T. ciliatum* exhibited 100% inhibition of HSV-1 at 20 μg/mL concentration by inhibiting the formation of plaque, resulting in a replication blockage [17]. 

**Table 6:** Antiviral activity of seagrasses.

Species	Extract/Active Compound	Virus	Inhibition	References
*P. oceanica*	MeOH/H_2_O 7:3 (balls extract)	H5N1	45% inhibition at 100 μg/mL	[25]
*T. ciliatum*	Methanolic extract	Hepatitis A (HAV) and herpes simplex (HSV-1)	100% inhibition at 20 μg/mL	[17]
*T. hemprichii*	Methanolic extract	HCV	50% inhibition at 23 μg/mL	[109]
*Zosteraceae family*	Polyphenol complex	Tick-borne encephalitis (TBE)	Suppressed accumulation of the pathogen in the cell culture at 100 μg/mL concentration.	[113]

### 3.7. Anti-Dengue Activity

Dengue fever is a global arboviral infection caused by four antigenically distinct dengue virus serotypes: DENV-1, DENV-2, DENV-3, and DENV-4 [114]. They are classified into RNA viruses that belong to the *Flavivirus* genus/*Flaviviridae* family [114]. The infection is endemic in more than 100 countries, particularly in Southeast Asia, the western Pacific region, and the Americas [115]. It is considered one of the most prevalent and widely spread diseases transmitted by female mosquito vectors. The primary vectors for the spread of this infection are *A. aegypti* and *A. albopictus*, which are mostly found in tropical and subtropical regions [115].

Dengue infection causes several clinical manifestations such as fever, headache, myalgia, rash, leukopenia, thrombocytopenia, and increased liver function [114]. Severe infections cause severe thrombocytopenia, hemorrhaging, and plasma leakage [114]. Until now, there are no protective vaccines against this infection, and their progression is inhibited by abolishing the mosquito vectors. Most of the current mosquito abatement programs are based on the use of larval insecticides [116]. Currently, active endeavors have been directed to the natural extracts of the botanical origins as potent compounds for mosquito larvae. There is no specific antiviral therapy for dengue infection; in fact, dengue fever is usually maintained through adequate hydration and fluid replacement therapy. [114]. Seagrasses produce secondary metabolites that have insect growth inhibitory activity, which can kill mosquito larvae. The underlying mechanism may involve the inhibition of *A. aegypti* larvae alterations in the spiracular valves of the siphon and anal papillae [117]. Studies on larvicidal activities from several seagrass extracts are shown in Table 7.

**Table 7:** Larvicidal activity of seagrasses.

Species	Extract/Active Compound	Mosquito	LC_50_ (μg/mL)	LC_90_ (μg/mL)	References
*C. serrulata* (leaves)	EtOH/water (3:1)	*A. aegypti*	0.0780	0.1675	[115]
	70% ethanol	*A. aegypti*	42.9	-	[116]
*E. acorodies*	Distilled water	*A. aegypti*	0.0852	0.1369	[117]
*H. ovalis*	Distilled water	*A. aegypti*	0.067	0.128	[117]
*H. pinifolia* (roots)	70% ethanol	*A. aegypti*	22.0	54.2	[116]
*S. isoetifolium* (leaves)	EtOH/water (3:1)	*A. aegypti*	0.0620	0.8970	[115]
*S. isoetifolium* (root)	EtOH/water (3:1)	*A. aegypti*	0.0604	0.9090	[115]
*T. hemprichii*	Ethanolic extract	*A. aegypti*	201.7	-	[116]
*T. testudinum* (leaves)	70% ethanol	*A. aegypti*	44.8	81.2	[116]

### 3.8. Lipid-Reducing Activity

Obesity is a disease associated with poor mental health outcomes and reduced quality of life, and it affects around 600 million people in the world [118]. Obesity is a major risk factor for cardiovascular diseases, diabetes, musculoskeletal disorders, and forms of cancer [118]. Obesity is caused by several factors, such as physical inactivity, a poor diet, and genetic susceptibility, which leads to the accumulation of fat in various body regions [118]. Large quantities of fatty acids from the diet must be transported as triglycerides to protect the body against their toxicity. Elevations in plasma triglyceride are the result of overproduction and impaired clearance of very low-density lipoproteins (VLDL) and chylomicrons, as well as the reduced expression of high-density lipoproteins (HDL) [119]. Elevated levels of VLDL and triglycerides, together with the reduction in HDL levels, lead to hyperlipidemia.

HDL cholesterol levels were significantly reduced in untreated diabetic mice and enhanced significantly in *T. hemprichii* extract-treated animals [120]. *T. hemprichii* ethanolic extract decreased both LDL and VLDL cholesterol levels in alloxan-induced diabetic mice [120]. Furthermore, ethyl acetate and methanolic extracts of *H. stipulacea* were tested using the zebrafish Nile red fat metabolism assay and showed IC_50_ values of 2.2 µg/mL and 1.2 µg/mL, respectively, after 48 h [21]. Its mechanism of action is through the inhibition of the acetyl-CoA carboxylase and PPARα agonists [21].

### 3.9. Antidiabetic Activity

Diabetes mellitus (DM) is a cluster of syndromes characterized by hyperglycemia; the altered metabolism of lipids, carbohydrates, and proteins; and an increased risk of complications from vascular diseases [121]. DM is a global health problem, and its incidence is increasing at an alarming rate throughout the world. Decreased physical activity, increasing obesity, stress, and changes in food consumption have been cited as reasons for the increasing diabetic prevalence in the past two decades [122]. The treatment of type 2 DM with oral hypoglycemic agents such as sulfonylurea and biguanides is associated with numerous side effects [121]. The major advantages of herbal medicine seem to be their good potential, low incidence of serious side effects, and low cost. Seagrasses contain many flavonoids and sterols/triterpenoids as their main constituents, which are known bioactive compounds for antidiabetic potential [123]. Flavonoids are also known to regenerate damaged β-cells in diabetic mice [123].

Compounds **52** and **53** isolated from *S. filiforme* and *T. testudinum* have potential for type 2 DM treatment by increasing GLUT4 translocation and expression [124,125]. *H. stipulacea* extracts at doses of 100 and 200 mg/kg/day demonstrated 9- and 13-fold increases in serum NO, respectively, compared to diabetic controls. Its mechanism of action was predicted as a result of the improvement of glucose uptake by the tissues through the restoration of liver GLUT-2 [126]. Moreover, *H. stipulacea* extracts ameliorated oxidative stress status generated by the free radicals and dyslipidemia under diabetic conditions [126]. Weight loss is one of the clinical features of DM due to adipocytes and muscle tissue degeneration, which make up for the energy lost from the body as the result of frequent urination and the conversion of glycogen to glucose. Alloxan-mediated bodyweight reduction was significantly reversed by the administration of *T. hemprichii* ethanolic extract for 15 days of treatment. The intraperitoneal administration of the extract resulted in a notable increase in body weight [120].

The methanolic extract of *H. beccari* exhibited a 50% inhibition of α-amylase and α-glucosidase at 270 µg/mL and 100 µg/mL, respectively [121]. It also regulated the glucose movement out of the cells and took up glucose by facilitating diffusion into the bloodstream, thus controlling post-postprandial glucose levels [121]. The same extract from *H. uninervis* reduced serum glucose levels in Streptozotocin-induced diabetic rats. The administration of 150 mg/kg *H. uninervis* extract decreased glucose levels by 24.8% after 6 h and exhibited a 52.5% reduction in glucose levels in the serum absorbed on the 18th day of administration at a dose of 150 mg/kg. Moreover, the administration of 250 mg/kg *H. uninervis* extract decreased glucose levels by 29.9% after 6 h and by 61.9% on the 18th day [123]. *P. oceanica* (L.) Delile hydroalcoholic leaves extracts exhibited strong in vitro activity against human serum albumin glycation, validating the recognized traditional antidiabetic role of these extracts. No advanced glycation end products were formed by incubating human serum albumin with glucose in the presence of 0.2 mg of dry extract for 72 h [127].

### 3.10. Hepatoprotective

The liver is a vital organ for survival, and it contributes to almost every metabolic function of the body, regulating homeostasis; it is also a frequent target for many toxicants [128]. In addition, the liver plays an important role in the storage of vitamins, iron, and copper, as well as in the detoxification of a large number of endogenous and exogenous substances [128]. Liver damage can include fatty liver, necrosis, cholestasis, hepatitis, and liver cirrhosis. Damage to the liver can be overcome by preventive (hepatoprotective) and curative (antihepatotoxic) efforts [129]. Conventional drug therapy for various liver damage diseases has limited efficacy and potentially adverse effects [130]. Treatment using extracts derived from natural resources is considered the best method to maintain liver function in the long term without significantly inducing toxic effects [130]. Liver cells containing various enzymes, such as SGOT and SGPT, are important for the diagnosis of liver damage because the enzyme is passed into the blood vessels [129]. Elevated enzyme activity may indicate the presence of liver disease [129].

The ethanolic extract of *T. hemprichii* exhibited hepatoprotective activity by lowering the levels of SGPT and SGOT in alloxan-induced diabetic mice [120]. Moreover, rats treated with higher doses of the *H. uninervis* methanolic extract (150 and 250 mg/kg) showed significant improvements in hepatic and renal function [123]. The administration of 280 mg/kg ethanolic extract of *C. rotundata* rhizome can significantly decrease SGPT and SGOT levels in paracetamol-induced rats [131]. The hydro-methanolic extract of *T. ciliatum* improved histopathological changes in the liver by inducing antioxidant defense enzymes superoxide dismutase (SOD); elevating GSH (non-enzymatic antioxidant glutathione); and reducing lipid peroxidation, nitric oxide (NO), alanine aminotransferase (ALT), and aspartate aminotransferase (AST) levels in thioacetamide (TAA)-induced liver failure [129]. The underlying mechanism is probably the stimulation of the Nrf2/ARE pathway, which results in the induction of antioxidant enzymes and the modulation of intracellular GSH-P in response to stress [129]. 

### 3.11. Anti-Aging Effects

Skin aging is a biochemical process resulting from intrinsic and extrinsic factors such as age, hormones, lifestyle, and exposure to UV [132]. The aging process takes effect in the epidermal and dermal layers, which are predominantly related to extracellular matrix (ECM) degradation. ECM consists of several enzymes, including matrix metalloproteinases (MMPs) and collagenase [132]. Collagen is the main constituent of the dermal matrix and is produced by fibroblasts. It is essential for skin tone and turgor, and it undergoes physiological turnover [133]. During skin aging, collagen degradation tends to overwhelm renewal, resulting in the formation of fine lines, wrinkles, and other alterations [133]. Hence, the maintenance of fibroblast function is a prerequisite for reducing skin aging. *P. oceanica* L. Delile ethanolic extract showed a significant increase in collagen production in fibroblasts exposed to 5 and 10 µg/mL and an increase in lipolysis in the concentration range of 10–200 µg/mL [133].

One of the manifestations of aging is hyperpigmentation, which occurs when the skin produces more melanin [132]. Tyrosinase is a rate-limiting enzyme for melanogenesis that converts tyrosine to melanin. Tyrosinase inhibitors play an important role as skin-lightening agents [132]. These inhibitors specifically interact with melanogenic cells and do not lead to side effects compared with other melanogenesis inhibitors [134]. The ethanolic extract of *P. oceanica* L. Delile also induced 20% tyrosinase inhibition at 5 µg/mL and 45% inhibition at 1000 µg/mL [133].

## 4. Bioactive Compounds from Seagrass under the Clinical Trial

Seagrasses share most features of their primary and secondary metabolism with terrestrial plants, since they are derived from land plants, which have secondarily recolonized marine habitats. In this review, we report some bioactive compounds isolated from seagrasses that have been tested in clinical trials, such as rutin (**2**), ferulic acid (**19**), quercetin (**20**), gallic acid (**23**), azelaic acid (**26**), lauric acid (**33**), and rosmarinic acid (**54**). However, to the best of our knowledge, there are no drugs commercially available that are isolated from seagrasses. 

A clinical trial of compound **2** for skin aging has been conducted by doing a double-blind clinical study in 40 subjects aged 30–50 years and divided into control and experimental groups. Compound **2** increased the mRNA expression of collagen, type I, and alpha 1 (COL1A1) and decreased the mRNA expression of matrix metallopeptidase 1 (MMP1) in HDFs. It was verified that ROS scavenging activity was stimulated by rutin in a dose-dependent manner. In addition, compound **2** exerted protective effects under oxidative stress conditions and increased skin elasticity while decreasing the length, area, and number of wrinkles [134].

Another clinical trial of compound **2** was a controlled study conducted on 53 type 2 diabetes patients randomized into three groups: 20 participants received rutin with vitamin C (group A), 20 received vitamin C (group B), and 13 received antidiabetic treatment only (group C). After eight weeks, significant reductions were observed in the % change of fasting blood glucose (FBG) of groups A and B versus group C. Vitamin C supplementation alone or with compound **2** significantly reduced the % change of FBG compared to controls but had no effect on HbA1c, FBG, TC, fasting insulin, and HOMA-IR or oxidative stress in T2DM patients [135].

Ferulic acid’s (**19**) ability to treat hyperlipidemia has been tested in a clinical trial. The study design was a randomized, double-blind, and placebo-controlled trial. Subjects with hyperlipidemia were randomly divided into two groups. The treatment group (n = 24) was given compound **19** (1000 mg daily), and the control group (n = 24) was provided with a placebo for six weeks. Compound **19** supplementation demonstrated a statistically significant decreases in total cholesterol (8.1%; *p* = 0.001), LDL-C (9.3%; *p* < 0.001), and triglyceride (12.1%; *p* = 0.049), as well as increased HDL-C (4.3%; *p* = 0.045) compared with the placebo. Compound **19** also significantly decreased the oxidative stress biomarker and the inflammatory markers [136].

Quercetin (**20**) has strong antioxidant, anti-inflammatory, immunomodulatory, and antiviral properties. It is also characterized by a very high safety profile and is exerted in animals and in humans. Like most other polyphenols, compound **20** has a very low rate of oral absorption, and its clinical use has been considered of modest utility. Compound **20** in a delivery-food grade system with sunflower phospholipids (Quercetin Phytosome^®^, QP) increases its oral absorption up to 20-fold. In the reported clinical trial (a randomized, controlled, and open-label study), a daily dose of 1000 mg of QP was investigated for 30 days in 152 COVID-19 outpatients to disclose its adjuvant effect in treating the early symptoms of the disease and preventing severe outcomes. The results revealed reductions in the frequency and length of hospitalization, the need for non-invasive oxygen therapy, the progression to intensive care units, and deaths. The results also confirmed the very high safety profile of compound **20** and suggested possible anti-fatigue and pro-appetite properties. QP is a safe agent and, when used in combination with standard care during the early stages of viral infection, could aid in improving the early symptoms and prevent the severity of COVID-19. Further research is needed to confirm these results [137].

The combination of 4% niacinamide + 1% gallic acid (**23**) + 1% lauric acid (**33**) can be used as an alternative topical treatment for acne vulgaris, which is a chronic inflammatory skin disease. In addition, this combination could be used to prevent resistance to topical antibiotics and side effects that might be caused by other skin disease treatments [138].

Azelaic acid (**26**) is known as an antioxidant agent. However, compound **26** was reported in a clinical trial for its anti-inflammatory activity. The results suggested that this new non-irritating product represents a valid therapeutic option for mild/moderate inflammatory rosacea. Furthermore, the evaluation of erythema changes was clearly defined by the instrumental evaluation of erythema degree by erythema-directed digital photography (EDDP) [139].

Previously, rosmarinic acid (**54**) was reported as an antioxidative agent. However, the capacity of compound **54** to treat AD has been investigated in clinical trials. A randomized placebo-controlled double-blind 24-week trial using *Melissa officinalis* extract richly containing compound **54** was carried out on patients with mild dementia due to AD to examine the safety and tolerability of this compound (500 mg daily), its clinical effects, and disease-related biomarker changes. There were no significant differences in cognitive measures; however, the mean Neuropsychiatric Inventory Questionnaire (NPI-Q) score improved by 0.5 points in the *M. officinalis* group and worsened by 0.7 points in the placebo group between the baseline and 24-week visit, indicating a significant difference. No significant differences were apparent in disease-related biomarkers between the groups. *M. officinalis* extract containing 500 mg of compound **54**, taken daily, was safe and well-tolerated by patients with mild dementia due to AD. The results suggest that compound **54** helps prevent the worsening of AD-related neuropsychiatric symptoms [140].

In this context, the indication of the percentage of the bioactive compounds’ abundance in the seagrasses identifies them as an alternative and valuable source of those compounds. However, to the best of our knowledge, there are no drugs commercially available that were isolated specifically from seagrasses. Moreover, a limitation of this study is the scarcity of reports about the bioavailability and pharmacokinetics of the bioactive compounds mentioned.

## 5. Conclusions

The objective of this review was to compile the most recent promising bioactivities of seagrass secondary metabolites and extracts on human health. The compounds described were organized on the basis of their bioactivities and, consequently, their chemical structure. The addressed biological compounds fall into four major classes: steroids, fatty acids, terpenes, and (mainly) polyphenols, including flavonoids, catechins, chalcones, phenylpropanoids, and phenylethanoids. Our efforts were intended to create a comprehensive collection of more than 50 natural products isolated from seagrasses that have been reported in the last decade, thus shedding light on the promising bioactive potential such as anticancer, antioxidant, anti-inflammatory, antimicrobial, antifungal, antiviral, anti-dengue, anti-dyslipidemia, antidiabetic, hepatoprotective, and anti-aging properties. A deep investigation of these effects and the discovery and characterization of metabolites derived from seagrasses could enable the preparation of innovative drugs, food supplements, and nutraceuticals for the management of several diseases and metabolic syndromes. Despite the huge interest in their ecological role, documented by around 30% of publisher papers in the last decade, the scientific community has not sufficiently addressed interesting aspects of the bioactive properties of seagrasses’ secondary metabolites. This overview provides the scientific bases for re-examining these metabolites with the latest available analytical techniques and the phytochemical investigation of the bioactive seagrass extracts, particularly with more applications for the isolation and structure elucidation. The advent of new analytical techniques, such as high-performance liquid chromatography coupled with tandem high-resolution mass spectrometry (UHPLC-HRMS/MS), can speed up the dereplication in comparative studies for the occurrence or absence of known compounds and improve efficiency in the discovery of new bioactive substances. 

Furthermore, after the preliminary screening to identify promising natural products derived from seagrasses, compounds should be evaluated for their safety and efficacy in animal models before being tested in clinical trials and used to develop drugs. A limitation of this study is the scarcity of reports about the bioavailability and pharmacokinetics of the addressed bioactive compounds. In addition, to the best of our knowledge, there are no commercially available bioactive compounds isolated from seagrass. Therefore, future studies should include the physical and chemical properties, bioavailability, and pharmacokinetic aspects of seagrasses’ secondary metabolites. Future studies should also determine the appropriate pharmaceutical form or drug delivery system to explore these marine angiosperms’ potential pharmacological applications. It is clear that natural products still have much potential value in the field of marine angiosperms.

## Data Availability

Not applicable.
